# Nonstationary time series forecasting using optimized-EVDHM-ARIMA for COVID-19

**DOI:** 10.3389/fdata.2023.1081639

**Published:** 2023-06-14

**Authors:** Suraj Singh Nagvanshi, Inderjeet Kaur, Charu Agarwal, Ashish Sharma

**Affiliations:** ^1^Department of Computer Science & Engineering, Ajay Kumar Garg Engineering College, Ghaziabad, Uttar Pradesh, India; ^2^Department of Computer Science & Engineering, Maharaja Agrasen Institute of Technology, New Delhi, India

**Keywords:** time-series, forecasting, COVID-19, optimized-ARIMA, optimized-EVDHM, PPT

## Abstract

The Coronavirus (COVID-19) outbreak swept the world, infected millions of people, and caused many deaths. Multiple COVID-19 variations have been discovered since the initial case in December 2019, indicating that COVID-19 is highly mutable. COVID-19 variation “XE” is the most current of all COVID-19 variants found in January 2022. It is vital to detect the virus transmission rate and forecast instances of infection to be prepared for all scenarios, prepare healthcare services, and avoid deaths. Time-series forecasting helps predict future infected cases and determine the virus transmission rate to make timely decisions. A forecasting model for nonstationary time series has been created in this paper. The model comprises an optimized EigenValue Decomposition of Hankel Matrix (EVDHM) and an optimized AutoRegressive Integrated Moving Average (ARIMA). The Phillips Perron Test (PPT) has been used to determine whether a time series is nonstationary. A time series has been decomposed into components using EVDHM, and each component has been forecasted using ARIMA. The final forecasts have been formed by combining the predicted values of each component. A Genetic Algorithm (GA) to select ARIMA parameters resulting in the lowest Akaike Information Criterion (AIC) values has been used to discover the best ARIMA parameters. Another genetic algorithm has been used to optimize the decomposition results of EVDHM that ensures the minimum nonstationarity and maximal utilization of eigenvalues for each decomposed component.

## 1. Introduction

Time series forecasting is a crucial area in machine learning, as it involves predicting future values of a variable or characteristic that is dependent on time and recorded at regular intervals. The ability to accurately forecast time series is essential in many fields, such as economics, finance, healthcare, transportation, and energy, to name a few. Time series forecasting helps organizations in projecting product demand, allocating resources efficiently, predicting maintenance schedules, and many other applications. Over the years, several approaches to time series forecasting have been proposed in the literature.

For instance, time series forecasting has been employed in various sectors, such as electricity prices (Contreras et al., 2003), wind power generation (Wan et al., 2014), electricity demand (Taylor and McSharry, 2007), traffic flow (Lippi et al., 2013), and taxi-passenger demand (Moreira-Matias et al., 2013). Moreover, time series forecasting has also been useful in predicting the spread of epidemic diseases such as Dengue (Appice et al., 2020) and Influenza (Saberian et al., 2014). These studies have demonstrated the effectiveness of time series forecasting in diverse fields and have inspired further research in this area.

Coronavirus Disease (COVID-19) is caused by the SARS-CoV-2 virus [Coronavirus disease (COVID-19) pandemic, 2021a]. COVID-19 was proclaimed a global pandemic on March 11th, 2020, after discovering the first known case in Wuhan, China [Coronavirus disease (COVID-19) pandemic, 2021b]. The illness has since spread around the globe, culminating in a pandemic. Various researchers have expressed interest in developing Artificial Intelligence based solutions to assist governments and enterprises in making decisions. The past COVID-19 forecasting investigations are summarized in Table 1.

**Table 1:** A summary of predicting models for Covid-19 forecasts.

**S.no**	**Year**	**Author**	**Investigated region(s)**	**Method(s) used**	**Accuracy measures**
1	2020	Rustam et al. (2020)	Australia, Canada, Algeria, and Afghanistan	Support Vector Regression (SVR), Linear regression (LR), Exponential Smoothing (ES), Least Absolute Shrinkage, Selection operator (LASSO), and Linear regression (LR)	Mean Square Error (MSE), Root Mean Square Error (RMSE), Mean Absolute error (MAE), *R*-square, Adjusted *R*-square
2	2020	Kumar and Susan (2020)	Global cases, Spain, Italy, India, France, Russia, Iran, UK, US, Turkey, Germany	ARIMA, PROPHET	MAE, RMSE, Root Relative Squared Error (RRSE), Mean Absolute Percentage Error (MAPE)
3	2020	Andreas et al. (2020)	Global cases	Curve fitting	*R*-square
4	2021	Dash et al. (2021)	Brazil, France, India, Russia, United Kingdom, US	ARIMA	RMSE, MAE, MAPE
5	2021	Satu et al. (2021)	Bangladesh and Global cases	PROPHET, LR, Polynomial-regression (PR), SVR, Multilayer Perceptron (MLP), and Polynomial-MLP (poly-MLP)	RMSE, R-squared
6	2021	Kurniawan and Kurniawan (2021)	Indonesia	Curve fitting	MSE
7	2020	Darapaneni et al. (2020)	India	ARIMA	R-square, Bayesian Information Criteria (BIC), Akaike's Information Criteria (AIC), and MSE
8	2021	Sharma et al. (2021)	India, USA, Brazil	ARIMA	RMSE
9	2020	Mustafa and Fareed (2020)	Iraq	ARIMA	MSE and MAE
10	2021	Kumar and Kaur (2021)	Delhi (India)	ARIMA, Gaussian Process Regression (GPR), LR, M5 Rule MLP, Support Vector Regression, Multi-Criteria Decision Making (MCDM), and Self-organized maps and fuzzy time series (SOMFTS)	Normalized-RMSE, Mean Magnitude of Forecasting Error (MMFE) Square root of the variance of the magnitude of residual errors (SdARE), The proportion of anticipated instances with a relative error magnitude of < 0.20
11	2021	Iqbal et al. (2021)	Pakistan	Long short-term memory (LSTM)	MAPE
12	2021	Zhan et al. (2021)	24 North American countries, 11 South American countries, 45 Asian countries, 46 European countries, 54 African countries, and 4 Oceanian countries	Gated Recurrent Unit (GRU), LSTM, Artificial Neural Network (ANN), Particle swarm optimization broad learning system (PSO- BLS)	R-square, MAE, RMSE

ARIMA is a time-series forecasting model that is one of the most often utilized approaches. ARIMA-based models are commonly employed in stationary time-series analysis (Wilson, 2016), although the standard ARIMA-based approach is inefficient in real-world settings for time series with nonstationary properties (Li and Chiang, 2013; Yang and Lin, 2016). EVDHM is a modern approach for nonstationary time series forecasting that may be combined with ARIMA (Sharma et al., 2021).

The COVID-19 pandemic has brought about an urgent need for accurate forecasting of the spread of the disease, to inform decision-making and public health interventions. As shown in Table 1, multiple studies have explored the use of machine learning (ML) and time series forecasting models in predicting the spread of COVID-19. These studies have highlighted the potential of ML and time series forecasting models in guiding decision-making and administrations. For instance, Rustam et al. (2020) employed a supervised ML model to predict the number of COVID-19 cases, deaths, and recoveries and discovered that exponential smoothing outperformed other models. Similarly, Kumar and Susan (2020) used ARIMA and Prophet time series forecasting models and found that the ARIMA model was more effective in forecasting COVID-19 prevalence. Andreas et al. (2020) proposed an improved mathematical forecasting framework based on ML and cloud computing that uses real-time data to accurately predict the progress of the curve. Satu et al. (2021) developed a web portal that provides real-time information on COVID-19 cases in Bangladesh and worldwide, including an ML-based short-term forecasting tool. Other researchers, such as Darapaneni et al. (2020) and Kurniawan and Kurniawan (2021), have also presented models for forecasting COVID-19 prevalence in Indonesia and India, respectively. Finally, Sharma et al. (2021) proposed a new method for time-series forecasting of nonstationary data using a combination of EVDHM and ARIMA models.

Although these studies have shown promising results, the models used by the researchers are not completely automated and require manual analysis of the data to provide inputs to the models. This makes it difficult for non-technical individuals to use these models, and analysis of the data can be time-consuming and prone to human error. To address this limitation, this paper proposes an optimized EVDHM approach combined with the ARIMA model for automated nonstationary time series forecasting.

The proposed model is fully automated and requires no manual input, which makes it easy, efficient, and time-saving for forecasting COVID-19 cases. This model can be used by non-technical individuals and is less prone to human error. The optimized EVDHM approach is used to capture the nonstationarity of the COVID-19 time series data, while the ARIMA model is used to capture the autocorrelation in the data. The proposed model is optimized using a grid search algorithm to select the best hyperparameters. The performance of the proposed model is evaluated using RMSE and compared with the performance of the traditional ARIMA model.

In conclusion, the proposed automated nonstationary time series forecasting model has the potential to provide accurate and timely predictions of the spread of COVID-19. The model is easy to use, efficient, and less prone to human error, and can be used by non-technical individuals.

The remainder of this article is structured as follows. Section 2 covers the datasets used in this study. Section 3 introduces EVDHM and ARIMA, Section 4 provides the proposed model and the stage outcomes, Section 5 compares EVDHM and Optimized EVDHM, and Section 6 summarizes the findings. Finally, section 7 brings the article to an end.

## 2. Dataset used

The data set for COVID-19 new cases in India has been utilized in this research. Data from January 22nd to May 10th, 2020, has been used for training, and data from the 11th to the 30th of May 2020 has been used for testing to analyze the proposed model. The data set has been given by Johns Hopkins University's Center for Systems Science and Engineering (CSSE) and is accessible online (CSSE, 2021).

## 3. Methods used

### 3.1. EVDHM

In linear algebra, the Hankel matrix is a square matrix with skew-diagonals that are constants. A Hankel matrix is represented as follows (Sharma and Pachori, 2017):


(1) $$A=\left[\begin{array}{ccccccc} A_{1} & A_{2} & A_{3} & . & . & . & A_{N}\\ A_{2} & A_{3} & \; & \; & \; & \; & .\\ A_{3} & \; & \; & \; & \; & \; & .\\ . & \; & \; & \; & \; & \; & .\\ . & \; & \; & \; & \; & \; & A_{2N-3}\\ . & \; & \; & \; & \; & A_{2N-3} & A_{2N-2}\\ A_{N} & . & . & . & A_{2N-3} & A_{2N-2} & A_{2N-1} \end{array}\right]$$

Eigenvalue Decomposition may be used to decompose a square matrix into its eigenvalues and eigenvectors. In terms of eigenvalues and eigenvectors, matrix *A* may be written as follows (Sharma and Pachori, 2017):


(2) $$\begin{array}{l} A=V\lambda V^{-1}\; \end{array}$$

*V*, λ, and *V*^−1^ are the eigenvector matrix, eigenvalue matrix, and inverse eigenvalue matrix, respectively. A stationary time series with statistical features such as mean and variance is either time-invariant or has a reasonable variation. Because of the significant variance, forecasting a nonstationary time series is often more difficult or complex than forecasting a stationary time series. Therefore, it is usually processed to reduce the time series' nonstationarity when working with nonstationary time series. EVDHM is an algorithm that can be utilized for analyzing nonstationary time series. It decomposes a time series into components with changing trends, noise, and oscillating patterns (Sharma and Pachori, 2017). Other applications of EVDHM include cardiovascular signal analysis (Sharma and Pachori, 2018b; Sharma et al., 2019), muscle signal analysis (Sharma et al., 2019), and complex data processing (Sharma and Pachori, 2018a).

A time-series *S*_t_ = *t*_1_, *t*_2_, *t*_3_,... *t*_2N − 1_ can be written as a Hankel matrix *H* of size *N* × *N* as follows (Sharma and Pachori, 2017):


(3) $$H=\left[\begin{array}{ccccccc} t_{1} & t_{2} & t_{3} & . & . & . & t_{N}\\ t_{2} & t_{3} & \; & \; & \; & \; & .\\ t_{3} & \; & \; & \; & \; & \; & .\\ . & \; & \; & \; & \; & \; & .\\ . & \; & \; & \; & \; & \; & t_{2N-3}\\ . & \; & \; & \; & \; & t_{2N-3} & t_{2N-2}\\ t_{N} & . & . & . & t_{2N-3} & t_{2N-2} & t_{2N-1} \end{array}\right]$$

Then *H* can be expressed as follows (Sharma and Pachori, 2017):


(4) $$\begin{array}{l} H=V_{s}\lambda V_{s}^{-1}\; \end{array}$$

where eigenvalue matrix λ can be expressed as follows (Sharma and Pachori, 2017).


(5) $$\text{λ}=\left[\begin{array}{ccccccc} {\text{λ}}_{1} & 0 & 0 & . & . & . & 0\\ 0 & {\text{λ}}_{2} & \; & \; & \; & \; & .\\ 0 & \; & \; & \; & \; & \; & .\\ . & \; & \; & \; & \; & \; & .\\ . & \; & \; & \; & \; & \; & 0\\ . & \; & \; & \; & \; & {\text{λ}}_{N-1} & 0\\ 0 & . & . & . & 0 & 0 & {\text{λ}}_{N} \end{array}\right]\;$$

All of the values in the eigenvalue matrix are zero, except for the diagonal elements, which have eigenvalues ranging from λ_1_ to λ_N_. The decomposition of the time series relies heavily on this eigenvalue matrix. It may be written as a sum of matrices with one or more distinct eigenvalues.


(6) $$\begin{array}{l} \lambda =\lambda_{1}+\lambda_{2}+\ldots +\lambda_{M} \end{array}$$

(6)

Now, *H* becomes


(7) $$\begin{array}{l} H=V_{s}\lambda_{1}V_{s}^{-1}+V_{s}\lambda_{2}V_{s}^{-1}+...+V_{s}\lambda_{M}V_{s}^{-1}\; \end{array}$$

Let,


(8) $$\begin{array}{l} H=H_{1}+H_{2}+\ldots +H_{M} \end{array}$$

The first decomposed component $S_{\text{t}}^{1}$ is computed using the mean of the skew-diagonal elements of the matrix *H*_1_. The remaining components are computed in the same way, using the *H*_i_ matrices *i* ϵ {2,3, ..., *M*}.

### 3.2. ARIMA

ARIMA comprises Autoregressive (AR), Moving Average (MA), and Integration (I) models combined. The Autoregressive (AR) model is a regression-based model whose current value is determined by previous values. The lagged forecast errors create the moving average (MA) model. It makes the next forecast based on prior errors. To model a nonstationary series, ARIMA employs differencing, which is represented by the letter I in ARIMA. An ARIMA model having parameters *p, d*, and *q*, if applied on time series *S*_t_ = {*s*_1_, *s*_2_, *s*_3_, …} then, it will be expressed as follows (Wilson, 2016):


(9) $$\begin{array}{l} \left(1-\overset{p}{\underset{n=1}{\sum }}\phi_{n}\beta^{n}\right){\left(1-\beta \right)}^{d}s_{t}=\theta^{0}+\left(1+\overset{q}{\underset{n=1}{\sum }}\theta^{n}\beta^{n}\right)e_{t} \end{array}$$

where *p* is the AR component's order and *q* is the MA component's order, *p* and *q* are positive integers. The backshift operator β is interpreted as β^n^ s_t_ = s_t−*n*_, where *d* is the needed degree of differencing to keep the time series stationary. The deterministic trend term is denoted by the number θ^0^. *e*_t_ is the white Gaussian noise with zero mean and $\sigma_{\text{e}}^{2}$ variance.

## 4. Optimized EVDHM and ARIMA based model

ARIMA does not work well with nonstationary time series. It has a parameter *d* that takes the lagged series to cope with nonstationarity in the time series. However, it cannot be estimated successfully for nonlinear time series (Sharma et al., 2021). Compared to the original series, the EVDHM decomposes the actual time series into numerous components with high stationarity. Because the components are stationary, this breakdown strategy helps decrease predicting errors since the components are stationary.

The EVDHM-ARIMA-based model has been used for time series forecasting (Sharma et al., 2021). In this work, an optimized EVDHM and ARIMA-based hybrid model has been used to create a time series forecasting model that is both efficient and automated. The Phillips Perron Test (PPT) test has been initially used to test for the stationarity of the time series. Then, EVDHM is applied to the time series in case of failure of PPT that results in the decomposed components. EVDHM employs a genetic algorithm to select the eigenvalues to decompose the series. The genetic algorithm aims to increase the number of eigenvalues selected while minimizing the series' nonstationarity.

The decomposition of a time series into components using EVDHM are completely reliant on the matrix λ and how its *N* eigenvalues are split among the matrices λ^1^, λ^2^, ... λ^M^ Eq. (7). The simplest approach is to divide *N* eigenvalues for *N* matrices to get *N* components, but in this case, all of the components will not be stationary, and the number of components will be large, causing the proposed model to take a long time to forecast because each component will be fitted to ARIMA for individual component forecasting. To deal with this, a genetic algorithm has been used. The goal of this GA is to distribute *N* eigenvalues among M matrices in such a way that the majority of the components are stationary and the number of components is as low as possible Eq. (6). The following parameters and their values have been used by this genetic algorithm number of iterations = 50, number of bits (n_bits) = *M* (number of Eigenvalues), population size = 100, crossover rate = 0.9, mutation rate = 1.0/(n_bits).

**Figure 1:**
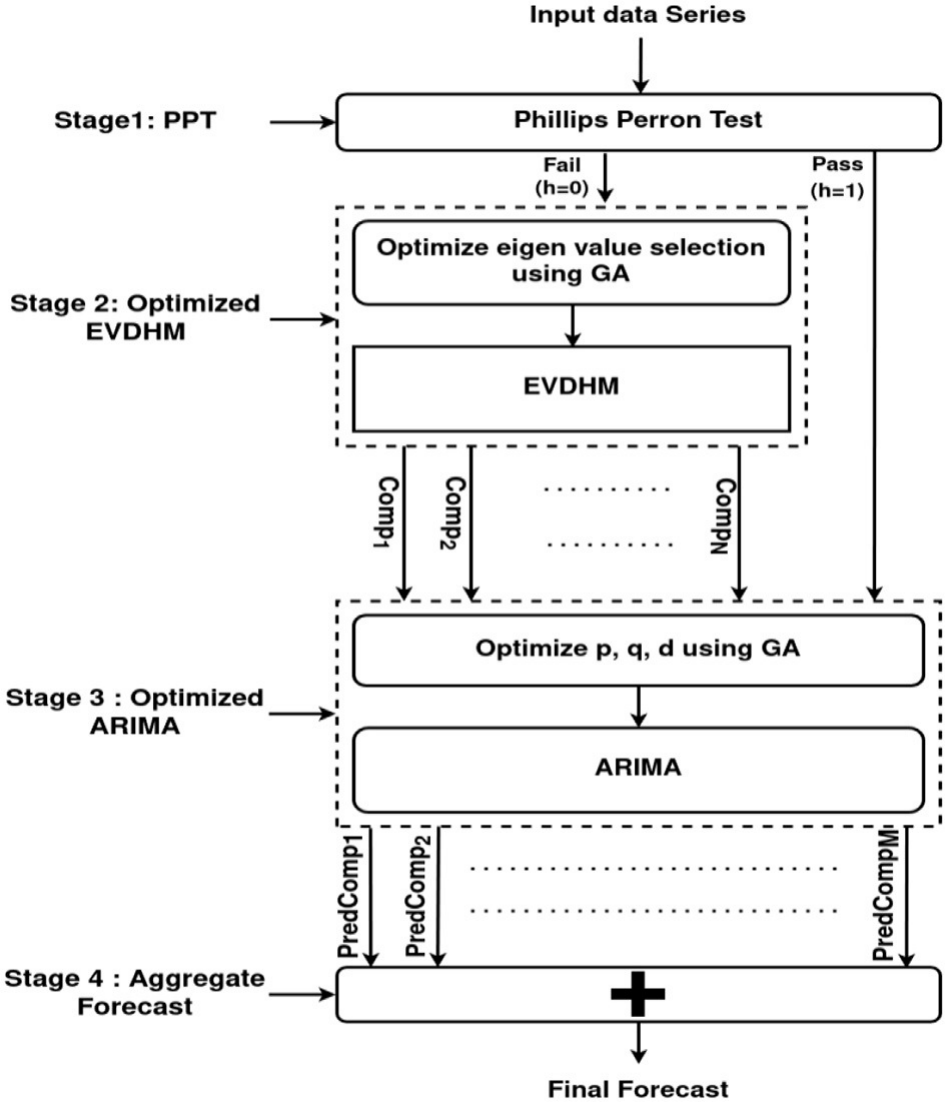
Model's block diagram based on O-EVDHM and O-ARIMA.

An *M*-digit binary number is used to represent individuals in this evolutionary method. For each binary digit, 1 and 0 denote the relevant eigenvalue selection or rejection. The goal of the genetic algorithm is to find the best possible combination of eigenvalues, including the maximum possible eigenvalues both at the same time that give a nonstationary component. The best possible combination of eigenvalues is picked after all iterations. With the remaining eigenvalues, this process is continued until no more stationary components are feasible. Because of the genetic algorithm, the generated components have a low count and are also stationary. Finally, the components are subjected to ARIMA, and ARIMA parameters are tuned using another genetic algorithm. Figure 1 depicts the proposed model's block diagram and also the stages of the optimized EVDHM and ARIMA-based model. The performance of the proposed model has been compared with the EVDHM-ARIMA-based model (Sharma et al., 2021). As indicated in Section 2, the data set from January 22nd to May 10th, 2020, is used for training, and the data set from May 11th to May 30th, 2020 is used for testing. The proposed model mainly consists of 4 stages PPT, Optimized EVDHM, Optimized ARIMA, and Aggregate forecast. A detailed explanation of each stage is given in the upcoming subsections.

### 4.1. Phillips Perron Test

The study employs the Phillips Perron test, which is a unit root test utilized to determine the stationarity of a time series. This test is distinct from other unit root tests such as the Dickey-Fuller test (DFT) and Augmented Dickey-Fuller test (ADFT) since it addresses serial correlation and heteroskedasticity in the errors. The automated features of PPT make it a better option than DFT and ADFT for this study (Patterson, 2011). In this study, the Boolean decision vector for the PPT is denoted by *h*, and the *p*-score is a left-tailed probability ranging from 0 to 1. The null hypothesis (*h* = 0) assumes that the underlying time series is non-stationary, while the alternative hypothesis (*h* = 1) asserts that it is stationary. A *p*-score < 0.05 leads to the rejection of the null hypothesis, indicating that the time series is nonstationary. Conversely, a *p*-score >0.05 leads to the acceptance of the null hypothesis and the rejection of the alternative hypothesis, indicating that the time series is stationary. The results of applying PPT to the training dataset in this study reveal that the dataset is nonstationary since the *h* and *p* scores are 0 and 1, respectively.

### 4.2. Optimized-EVDHM

**Figure 2:**
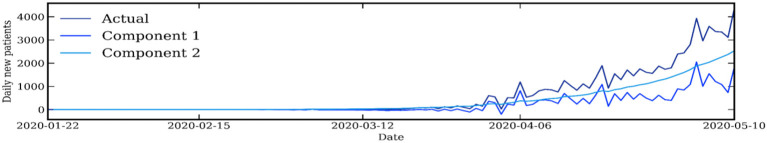
Trend (Component 1) and Variability (Component 2) of the actual series.

EVDHM transforms a data series into a Hankel matrix *H* and then decomposes the Hankel matrix into its eigenvalues and eigenvectors, as shown in Eq. (4). The eigenvalue matrix λ is decomposed into *M* matrices such that each matrix has one or more distinct eigenvalues of the matrix *H*. For an effective and efficient decomposition of a data series, the decomposed components should be less in count, and all the components should be stationary. The number of components should be less because ARIMA is applied to each component. Therefore, a high number of components will increase the running time of ARIMA. Moreover, the stationarity of the component will help the ARIMA for better predictions. A genetic algorithm has been used in the proposed model to decompose the eigenvalue matrix λ into λ_1_, λ_2_, . . ., λ_M_ matrices. The objective of this genetic algorithm is to select the eigenvalues of λ for λ_1_ such that it will try to maximize the eigenvalues utilization and minimize the nonstationarity of the component formed with λ_1_. Similarly, subsequent λ's are calculated. The resultant two components and the actual data series are shown in Figure 2.

### 4.3. Optimized ARIMA

ARIMA needs three parameters: *p, q*, and *d* for each decomposed component to fit the model. A genetic algorithm has been used in this research to automate parameter selection. The genetic algorithm (GA) selects the model's variables *p, q*, and *d*, having the lowest Akaike information criteria (AIC) value. The AIC is a mathematical tool for determining if a model sufficiently fits the data. Generally, the best fitting models are considered good. Therefore, the GA has been set to use 0–5 for all three parameters to find out the best set of parameters with the lowest AIC value. The derived parameters for each component have been fitted into the ARIMA model, and the fitted model was used to anticipate future values from May 10th through May 30th, 2020.

### 4.4. Aggregate forecast

**Figure 3:**
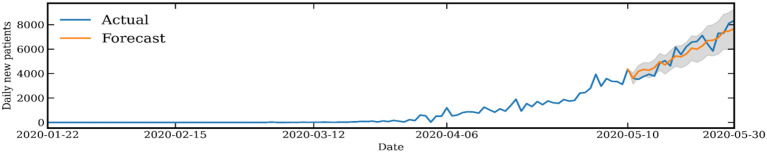
Final forecast from May 11, 2020 to May 30, 2020.

The ARIMA's separate component predictions have been combined to get the final projection. Figure 3 depicts the final forecast.

### 4.5. Performance of O-EVDHM

In this study, the performance of the Optimized EVDHM approach has been evaluated by calculating the sum of squared (SS) scores for all the decomposed components. The SS score is an important metric that measures the strength of the decomposition of the time series. The formula for calculating the SS score has been given in Eq. (10).


(10) $$\begin{array}{l} SS=\overset{n}{\underset{i=1}{\sum }}{\left(t_{i}\right)}^{2} \end{array}$$

where *t*_i_ is an *n*-sample data series with *i* being a positive integer less than or equal to *n*.

The SS scores for the decomposed components have been presented in Table 2. From the table, it can be observed that the SS score for the main signal is 1.47 × 10^8^, while the SS values for C_1_ and C_2_ are 2.40 × 10^7^ and 5.91 × 10^7^, respectively. The stationary component C_1_ captures the variability of the actual series, whereas the trend component C_2_ is nonstationary. Therefore, no further decomposition is required beyond C_2_.

**Table 2:** *h*-score, *p*-score, and SS score of decomposed components.

	**EVDHM (first iteration)**										**Optimized EVDHM**	
**Components**	**C**_1_	**C**_2_	**C**_3_	**C**_4_	**C**_5_	**C**_6_	**C**_7_	**C**_8_	**C**_9_	**C**_10_	**C**_1_	**C**_2_
*h* score	0	0	1	1	1	1	1	1	1	1	1	0
*p* score	0.9990	0.0764	0.0010	0.0010	0.0010	0.0010	0.0010	0.0010	0.0010	0.0010	0.04114	1
SS score	1.46 × 10^8^	7.5 10^5^	7.64 × 10^5^	4.6 × 10^5^	1.09 × 10^5^	8.03 × 10^4^	1.07 × 10^5^	6.38 × 10^4^	2.5 × 10^4^	9.6 × 10^3^	2.40 × 10^7^	5.91 × 10^7^

The performance of the proposed approach has been further assessed by comparing it with the EVDHM-based approach. As mentioned earlier, the EVDHM-based approach decomposes the time series into 10 components. The SS score of the trend component C_1_ calculated using the EVDHM technique is 1.46 × 10^8^, which is much higher than the trend component C_2_ calculated using the Optimized EVDHM method, 5.91 × 10^7^. This indicates that the Optimized EVDHM approach is more successful in decomposing the actual nonstationary series.

To evaluate the forecasting accuracy of the proposed approach, the RMSE has been calculated for both the EVDHM-based ARIMA model and the Optimized EVDHM-based ARIMA model. The RMSE for the EVDHM-based ARIMA model is 702.6, whereas the RMSE for the Optimized EVDHM-based ARIMA model is 538, which is significantly lower.

## 5. Comparison of EVDHM and optimized EVDHM

The Optimized EVDHM-based decomposition approach was developed to address the limitations of the EVDHM method used in (Sharma et al., 2021). For measuring the performance of the Optimized EVDHM method, it was compared to the EVDHM method in terms of *h*-score, *p*-value, and SS score for the decomposed components of the actual series.

Table 2 shows the comparison of the results. After the first iteration, the EVDHM decomposes the actual series into ten components C_1_-C_10_, as shown in Table 2. Component C_1_ has a trend, whereas the remaining components, C_2_-C_10_, have variability. The SS score of the trend component C_1_ calculated using the EVDHM technique is 1.46 × 10^8^, which is much higher than the trend component C_2_ calculated using the Optimized EVDHM method, 5.91 × 10^7^. This indicates that the Optimized EVDHM is more successful in decomposing actual nonstationary series.

Moreover, after the first iteration, the EVDHM method resulted in a total of 10 components, while the Optimized EVDHM method yielded only two. This reduction in the number of components implies that the Optimized EVDHM method can effectively and efficiently decompose nonstationary time series. In addition, the decomposition using the Optimized EVDHM does not require several iterations, making it a more suitable approach for nonstationary time series.

Overall, the results demonstrate that the Optimized EVDHM-based decomposition approach outperforms the EVDHM method in efficiency and effectiveness in decomposing nonstationary time series. This improvement is attributed to the use of a genetic algorithm in the process of selecting the eigenvalues best suited for each decomposition, which optimizes the decomposition process and yields better results. As such, the Optimized EVDHM method holds great promise for future applications in various fields, including finance, healthcare, and environmental studies, where nonstationary time series are ubiquitous.

## 6. Results and discussions

This paper presents an Optimized EVDHM and ARIMA-based time series forecasting model used to anticipate the COVID-19 Indian cases. As mentioned in section 2, 109 days of data were utilized for training the proposed model from January 22nd to May 10th, 2020. Forecasting has been done for the following 20 days till May 30th, as indicated in Figure 3. The blue line in Figure 3 depicts the actual data series, while the red line depicts predictions from May 11th to May 30th, 2020. The gray region represents the 95% percent confidence interval. The root means squared error (RMSE) for the anticipated values has been determined to assess the suggested model's performance.


(11) $$\begin{array}{l} RMSE=\sqrt{\frac{1}{n}\overset{n}{\underset{i=1}{\sum }}\left(P_{i}-A_{i}\right)}\; \end{array}$$

*P* and *A* are the series' predicted and observed values, respectively. The RMSE of the suggested model is 538 for the 20 days of projected values, but the RMSE of the EVDHM-ARIMA-based model is 702.6, which is much higher than the RMSE of the proposed model.

## 7. Conclusions

In conclusion, the proposed Optimized EVDHM and ARIMA-based approach for time series forecasting is demonstrated to be effective in predicting new cases of COVID-19. The numerical comparison shows that the Optimized EVDHM-based ARIMA model outperforms the EVDHM-based ARIMA model with an RMSE of 538, indicating the practical significance of this study. The approach utilizes a genetic algorithm-based approach for decomposing nonstationary time series into its constituent components, followed by the application of ARIMA for forecasting. The proposed technique can be applied to various signals in the future, such as power load, sales forecasts, and inventory research, among others, making it a versatile tool for time series forecasting.

The points of innovation in this study include the use of genetic algorithms for optimizing the EVDHM decomposition method, which leads to more efficient and effective decomposition of nonstationary time series. The proposed approach also utilizes ARIMA for forecasting, which is a widely used and reliable method for time series forecasting.

However, there are some current shortcomings in this study. The proposed approach has only been tested on COVID-19 data, and its performance on other datasets needs to be evaluated. Additionally, the proposed approach can only be applied to univariate time series, and its extension to multivariate time series remains an area for future research. Nonetheless, the proposed Optimized EVDHM and ARIMA-based approach demonstrates promising results and can be considered a valuable addition to the existing literature on time series analysis and forecasting.

## Data availability statement

Publicly available datasets were analyzed in this study. This data can be found at: https://github.com/CSSEGISandData/COVID-19.

## Author contributions

All authors listed have made a substantial, direct, and intellectual contribution to the work and approved it for publication.
